# The evolution of income-related inequalities in healthcare utilisation in Indonesia, 1993–2014

**DOI:** 10.1371/journal.pone.0218519

**Published:** 2019-06-25

**Authors:** Joko Mulyanto, Dionne S. Kringos, Anton E. Kunst

**Affiliations:** 1 Department of Public Health, Amsterdam UMC, University of Amsterdam, Amsterdam Public Health Research Institute, Amsterdam, the Netherlands; 2 Department of Public Health and Community Medicine, Faculty of Medicine, Universitas Jenderal Soedirman, Purwokerto, Indonesia; Institute of Economic Growth, INDIA

## Abstract

**Objective:**

While the major policy changes in the Indonesian healthcare system over the last 25 years have been well documented, less is known about the accompanying changes in inequalities in healthcare utilisation during this period. Our study aimed to describe the trends in income-related inequalities in healthcare utilisation in Indonesia during the period 1993–2014.

**Methods:**

A repeated cross-sectional study was conducted using data from the Indonesian Family Life Surveys from 1993, 2000, 2007, and 2014. We measured outpatient and inpatient healthcare utilisation in public and private provider as well as the overall utilisation. Standardised prevalence rate and relative index of inequality (RII) were used to measure the extent of inequalities in healthcare utilisation by income level (income-related inequalities).

**Results:**

Relatively large income-related inequalities were observed in the utilisation of private outpatient care and public and private inpatient care in 1993. Income-related inequalities in public and private outpatient care utilisation decreased between 1993 and 2007 but increased in 2014. Income-related inequalities in public and private inpatient care utilisation continued to decrease between 1993 and 2014. The largest decrease was observed in private inpatient care utilisation.

**Conclusion:**

Income-related inequalities in all types of healthcare utilisation decreased until 2007. This trend continued until 2014 only for public and private inpatient care utilisation. This phenomenon may be explained by the changes to the healthcare system (e.g. expansion of the government health insurance programme and health sector decentralisation), which coincided with the changes in inequalities in healthcare utilisation in Indonesia.

## Introduction

The magnitude of income-related inequalities in healthcare utilisation has been well documented, particularly in high-income countries [1, 2]. Many studies have focused on individual-level factors such as education and health-seeking behaviour to explain these inequalities [3–5]. Much less attention has been given to assessing how contextual factors contribute to these inequalities, which is particularly important for health policy evaluation and development.

Studies in Southern Europe and Finland have shown that contextual changes (e.g. decentralisation of the healthcare system both in terms of financing and health services delivery, and the introduction of co-payments) can contribute to changes in income-related inequalities in healthcare utilisation [6–9]. Studies in China and Brazil have shown that removing financial barriers to healthcare by implementing national health insurance (NHI) contributes to the reductions in income-related inequalities in healthcare utilisation over time, and other health system issues (e.g. unequal distribution of healthcare resources and privatisation) are also likely to play significant roles in the changes of income-related inequalities in healthcare utilisation [4, 10].

Although reducing inequalities in healthcare utilisation have been a global issue for some decades, this has only recently become a subject of research in Indonesia. Previous studies have shown that relatively large socioeconomic inequalities exist in Indonesia, particularly in maternal and child healthcare and dental care [11–13]. A more recent study has shown that Indonesia has large socioeconomic inequalities in general healthcare utilisation, particularly in secondary care utilisation. This study showed that after NHI was implemented in Indonesia, inequalities decreased only in private outpatient care [14]. However, these studies were conducted either at a specific point of time or during a limited period, providing limited insight into the course and nature of inequalities in healthcare utilisation in Indonesia over the last 25 years.

This period is particularly important in light of the critical changes that took place in the country’s healthcare system during this period. In Table 1, we provide a summary of the major changes to the Indonesian healthcare system from the early 1990s through 2014. In the early 1990s, the healthcare system was focused on public health interventions, particularly for maternal and child healthcare and infectious diseases. The government left the provision of personal healthcare to the market, and only civil servants were covered by government health insurance scheme. Healthcare was provided mainly by public healthcare providers, who received a limited subsidy from the government. Private hospitals were available only in the big cities to meet the demands of high socioeconomic status (SES) groups.[15]

**Table 1:** Milestones of the Indonesian healthcare system from the early 1990s through 2014 [15–17].

Period	Healthcare system features
The early 1990s	• Health sector development focus was on public health interventions; healthcare provision was left to the market. • No government health insurance programme for the poor, only for government employees. • Public dominance in healthcare delivery, with limited subsidies. • Limited number of private hospitals, mostly located in big cities.
1997	• Major economic crisis led to the introduction of a social safety net for healthcare (JPS-BK)
1999	• Expansion of JPS-BK into the government health insurance for the poor programme (ASKESKIN)^a^.
2001	• Health sector decentralisation at the district level. • Increasing number and role of private healthcare providers.
2004	• Expansion of the ASKESKIN^a^ programme coverage into the near-poor population (JAMKESMAS)^b^.
2007–2013	• Rapid increase in JAMKESMAS^b^ coverage, but mistargeted the enrolment of beneficiaries (excess demand). • The continuing decline of public investment in healthcare infrastructure. • Large interregional variation in healthcare infrastructure due to health sector decentralisation.
2014	• Introduction of the national health insurance (JKN) programme (2014), which aims to achieve universal coverage by 2019.

^a^ Government health insurance programme for the poor.

^b^ The expansion of government health insurance programme for the poor into the near-poor population.

The major economic crisis in 1997 led to decreased healthcare utilisation in all SES groups, and to the introduction of a social safety net in healthcare (known as JPS-BK) to buffer the impact of the economic crisis for lower SES groups. In this programme, the government allocated a special budget to reimburse the medical bills of those from lower SES groups who received healthcare from public primary healthcare centres or public hospitals. The Ministry of Health distributed the budget to the district health offices, which were responsible for covering the medical bills of lower SES group in their districts [15]. The government expanded the JPS-BK programme into the government health insurance programme for the poor (known as ASKESKIN) in 1999. Starting in 2001, there was a significant change to the decentralisation of the health sector at the district level. In 2004, ASKESKIN became the JAMKESMAS programme, which expanded the coverage of the government health insurance programme to the near-poor population [15]. Introducing the ASKESKIN and JAMSKESMAS programmes were effective, and increased healthcare utilisation among the poor, particularly for outpatient care [16, 17]. The next milestone was reached in 2014 with the introduction of the NHI programme, which aims to achieve universal coverage by 2019 [15].

Our study aimed to historically describe trends in income-related inequalities in healthcare utilisation in Indonesia during the period 1993–2014. In particular, we aimed to measure changes over time in absolute and relative income-related inequalities in the utilisation of public outpatient care, private outpatient care, public inpatient care, and private inpatient care as well as the overall utilisation. The results will be discussed within the context of the changes to the Indonesian health care system over the last 25 years.

## Methods

### Study design and population

A secondary analysis was conducted using repeated cross-sectional studies from four waves of the Indonesian Family Life Survey (IFLS): 1993, 2000, 2007, and 2014. The IFLS is an ongoing longitudinal household survey conducted by the RAND Corporation (USA). The IFLS was approved by the relevant ethical review committees in the United States and Indonesia. The data are publicly accessible through RAND’s website, and other details on the IFLS have been published elsewhere [18]. The IFLS collected data using stratified sampling from 13 Indonesian provinces, including the five provinces in Java island (inhabited by 57% of Indonesian population), four major provinces in Sumatra island, and four provinces represented other major island groups. These 13 provinces represented around 83% of the Indonesian population [18].

The present study made use of data from adult individuals aged 15 and older with complete data for all study variables. Sample size ranged from 14,202 individuals in the IFLS1 (1993) to 42,300 individuals in the IFSL5 (2014). The number of respondents increased over time because the following surveys included new respondents who had been part of another household in the previous survey but who were now part of a new household (e.g. due to marriage). Because we used a cross-sectional approach in our data analysis, we were able to include these new respondents.

### Measures

The level of household consumption was used as a proxy for income. In developing countries, consumption is considered the most valid direct measurement of income or household wealth [19]. This measure at household level included food, non-food consumables, durable goods, spending on education, and housing. These amounts were aggregated and adjusted into a monthly estimate, which was adjusted to household size to account for economies of scale. Geographical differences in purchasing parity were also adjusted for, using Jakarta’s poverty line as a reference. Income measurement for the different areas was adjusted, taking into account variations in poverty lines by province, as well as urban versus rural location of residence. The poverty lines were obtained from the Indonesian Central Bureau of Statistics; the households were grouped into quintiles based on these income measures. As rich households will use on average a smaller part of their income on food consumption, the use of consumption only as a proxy of income may underestimate the relative income of the rich households. Our analysis showed a strong correlation between food consumption and total consumption. Moreover, the proportion of food consumption of part of total consumption was between 20 and 23.5% for all income quintiles except the richest (14.2). This supports the use of household consumption as a proxy of income in our analysis (S1 Table).

We used self-reported outpatient and inpatient care utilisation on the IFLS. The recall period for outpatient care utilisation was four weeks, and 12 months for inpatient care. We measured overall healthcare utilisation both for outpatient and inpatient care and further distinguished healthcare utilisation based on the type of healthcare, which included public outpatient care, private outpatient care, public inpatient care, and private inpatient care. Self-assessed health (SAH) was used as a proxy for healthcare need. SAH is a health status measurement applicable to different socioeconomic groups (unlike chronic disease prevalence, for example). SAH data were obtained from the IFLS based on responses to the question, ‘*In general*, *how is your health*?’ The four response categories were ‘*very healthy*’, ‘*somewhat healthy*’, ‘*somewhat unhealthy*’, and ‘*very unhealthy’*.

For all respondents, we measured their urban or rural locations and provinces of residence using IFLS data. These geographical variables were included in order to control for confounding by geography. We regrouped the provinces into two groups (Java & Bali versus others) based on the similarity in the socioeconomic and cultural background. Survey year (1993, 2000, 2007, 2014) was used as a variable in the pooled-data analysis to measure the trends.

### Data analysis

To describe variations in healthcare utilisation between income groups, we calculated the prevalence rates for each type of healthcare by income quintile. Prevalence rates were measured as the number of cases per 100 persons; these rates were age and sex standardised using the direct method, with the total survey population as the standard population. Then, the rate difference and rate ratio were calculated based on the standardised prevalence rate of the two lowest income groups combined and the two highest income groups combined, respectively. Rate difference was calculated by subtracting the prevalence rate of high-income groups from that of the low-income groups, while rate ratio was calculated by dividing the prevalence rate of high-income groups by that of low-income groups [20].

The relative index of inequality (RII) was used to provide a more comprehensive estimate of the magnitude of income-related inequalities in healthcare utilisation. The RII is a regression-based index that assesses the probability of healthcare utilisation in relation to the relative hierarchical position of every individual within the income hierarchy. A higher RII indicates a stronger association between this hierarchical position and health care utilisation. This implies a greater utilisation difference between the higher and lower income groups: RII = 1 indicates equality, RII < 1 indicates inequality with higher utilisation among lower-income groups, and RII > 1 indicates inequality with higher utilisation among higher income groups. The RII is a valid health inequality measure for facilitating comparisons across diverse populations and outcomes [21]. The regression model was adjusted for age and sex, and in the final model was also adjusted for healthcare need by controlling for SAH and geographical differences. We performed post-estimation diagnostics of residuals to check the fit of the regression models. We found that outliers and single cases could not have strongly influenced the model fits and estimates.

We used pooled-data analysis to measure trends in healthcare inequalities. All subjects across the different waves of the survey were pooled into a single dataset. When calculating the RII, we included the years of the survey in our final model. We included the interaction variable between survey year and income level. The p-value for interaction was interpreted as the trends in healthcare inequality measurement. A similar post-estimation diagnostic was also applied in the pooled-data regression. To correct for attrition and oversampling, we weighted our study sample with individual weights provided by the IFLS. After weighting, the estimates were representative of the Indonesian population for each survey year.

## Results

In all income quintiles, both sample size and income levels increased considerably between different waves of the survey over time (Table 2). During the period 1993–2014, the lowest income group experienced a larger income increase compared with the other income groups. During this period, the ratio of median income between the highest and lowest income groups decreased. The proportion of male and female respondents remained stable, with a slightly larger proportion of women. The proportion of respondents from the oldest age category (60 and older) increased slightly over time. SAH status trends showed increases in the proportion of respondents in both the lowest (unhealthy) and the highest (very healthy) categories. Geographically, while the proportion of respondents living in urban areas gradually increased, the proportion of people living in Java and Bali and other places remained stable.

**Table 2:** Sample sizes and characteristics of the samples.

	1993		2000		2007		2014	
	N = 14,202		N = 25,322		N = 30,182		N = 42,300	
	n	%	n	%	n	%	n	%
**Sex**								
Male	6382	44.9	12309	48.6	14793	49.0	20473	48.4
Female	7820	55.1	13013	41.4	15389	51.0	21827	51.6
**Age**								
15–30	4264	30.1	9881	39.0	10977	36.4	12493	29.5
31–45	5016	35.3	8061	31.8	9010	29.8	14084	33.3
46–60	3372	23.7	4814	19.0	6724	22.3	10316	24.4
> 60	1550	10.9	2566	10.2	3471	11.5	5407	12.8
Income*								
1st quintiles (poorest)	18.47	20	95.22	20	289.10	20	987.00	20
2nd quintiles	31.27	20	151.13	20	455.99	20	1521.50	20
3rd quintiles	45.86	20	211.36	20	633.58	20	2008.03	20
4th quintiles	69.16	20	303.97	20	905.96	20	2981.60	20
5th quintiles (richest)	131.38	20	589.22	20	1615.32	20	5193.73	20
**Self-assessed health**								
Very healthy	2552	18.0	2042	8.1	3306	11.0	8164	19.3
Somewhat healthy	10117	71.2	20087	79.3	22715	75.3	24867	58.8
Somewhat unhealthy	1461	10.3	3142	12.4	4057	13.4	8505	20.1
Unhealthy	71	0.5	50	0.2	104	0.3	764	1.8
**Residence**								
Urban	4805	33.8	11304	44.6	13819	45.8	22141	52.3
Rural	9397	66.2	14018	55.4	16363	54.2	20159	47.7
**Province**								
Java & Bali	10647	75.0	19481	76.9	22531	74.7	31813	75.2
Others	3555	25.0	5841	23.1	7651	25.3	10487	24.8

* Median income of each quintile in thousands of Indonesian rupiahs (IDR).

**Fig 1:**
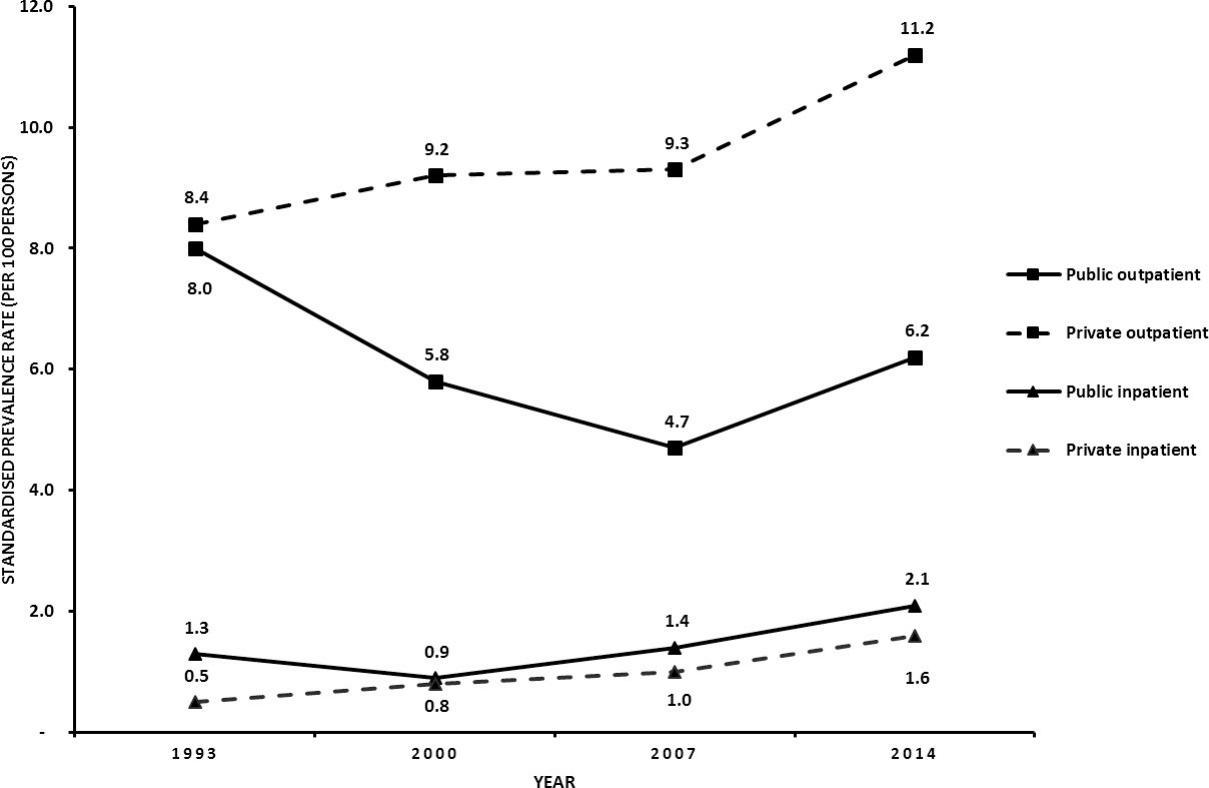
The standardised prevalence rate of healthcare utilisation for all types of healthcare, 1993–2014.

Fig 1 shows the trends in all types of healthcare utilisation in Indonesia. In 1993, public and private outpatient care had similar utilisation levels. However, private outpatient care utilisation continued to increase until 2014, while public outpatient care utilisation continued to decrease until 2007, with a slight increase in 2014. This resulted in much greater private outpatient care utilisation (11.2 per 100 persons) compared with public outpatient care utilisation (6.2 per 100 persons) in 2014.

In terms of inpatient care utilisation, in 1993 we found that utilisation levels for public inpatient care were much higher than those for private inpatient care. Although both public and private inpatient care utilisation continued to increase until 2014, private inpatient care utilisation increased more steadily. However, utilisation levels for public inpatient care were still higher (2.1 per 100 persons) than those for private inpatient care (1.6 per 100 persons) in 2014.

**Fig 2:**
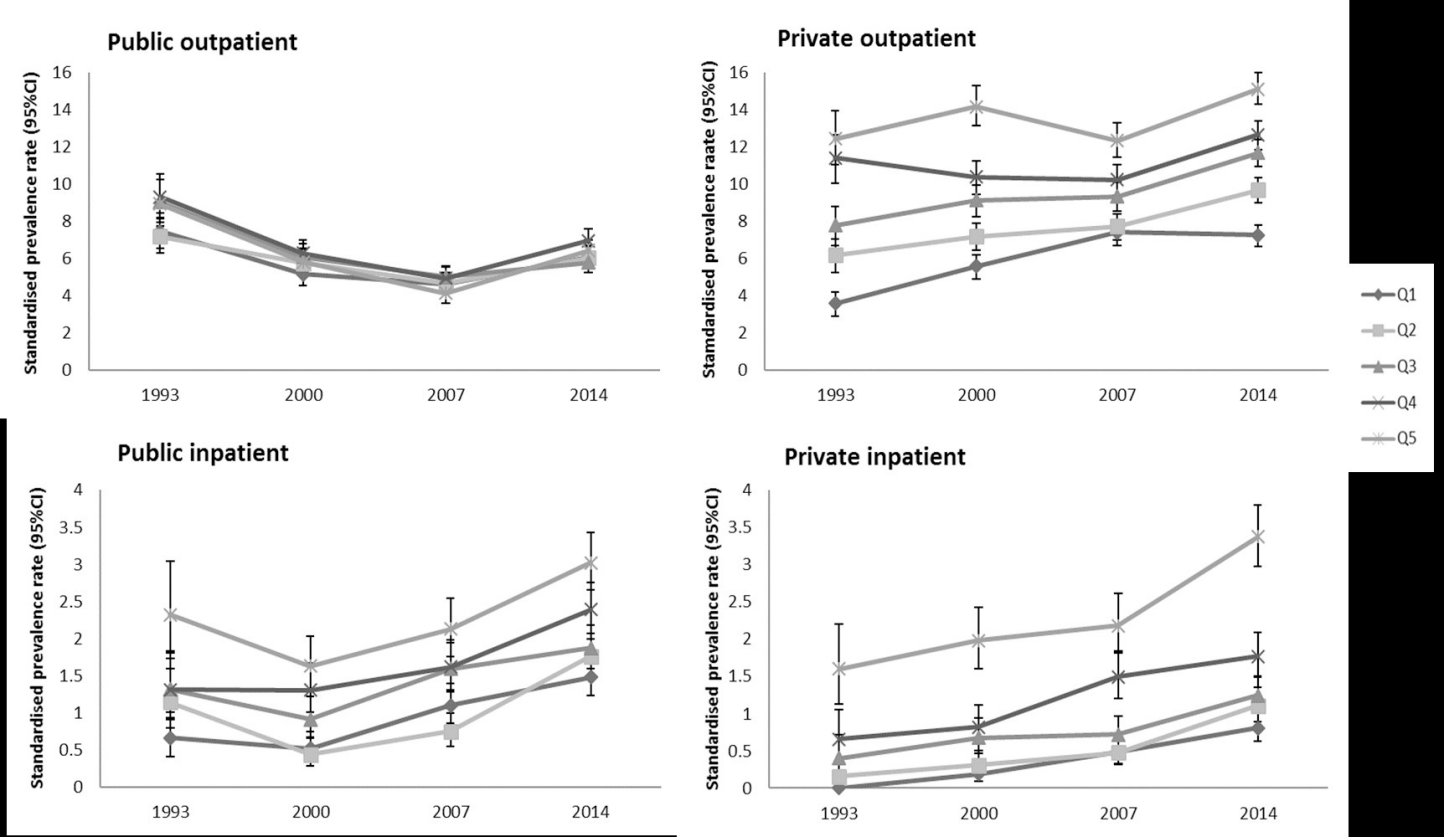
Standardised prevalence rate (95% CI) of healthcare utilisation by income quintiles, 1993–2014. Prevalence rate standardised by age and sex to total population, per 100 persons. Q1: first quintile (poorest); Q2: second quintile; Q3: third quintile; Q4: fourth quintile; Q5; fifth quintile (richest).

In 1993, the magnitude of income-related inequalities varied between the types of healthcare (Fig 2, Table 3). Private outpatient care had larger inequalities, with RII 6.18 (95% confidence interval (CI): 4.88–7.92) compared with public outpatient care, with RII 1.55 (95% CI: 1.24–1.95). The largest inequalities were found in private inpatient care, with RII 37.26 (95% CI: 11.81–117.60). This could be attributed to the lowest income group’s low (virtually non-existent) utilisation of private inpatient care. Public inpatient care had much smaller inequalities compared with private inpatient care, with RII 3.61 (95% CI: 2.05–6.34).

**Table 3:** Trends in absolute and relative income-related inequalities in healthcare utilisation, 1993–2014.

			1993		2000		2007		2014		p-value
Public outpatient	SPR (95% CI)	Highest 2 quintiles	8.92	(8.10–9.81)	6.44	(5.92–6.99)	4.76	(4.35–5.19)	6.70	(6.31–7.12)	
		Lowest 2 quintiles	7.08	(6.45–7.75)	5.71	(5.24–6.21)	4.76	(4.38–5.17)	6.06	(5.69–6.44)	
		Rate difference	1.85		0.73		-0.01		0.65		
		Rate ratio	1.26		1.13		1.00		1.11		
	RII (95% CI)	Adjusted to age, sex	1.44	(1.17–1.77)	1.14	(0.95–1.36)	0.94	(0.78–1.13)	1.19	(1.03–1.36)	< 0.01
		Adjusted to age, sex, need	1.46	(1.19–1.81)	1.14	(0.95–1.37)	0.95	(0.78–1.14)	1.22	(1.06–1.40)	< 0.01
		Adjusted to age, sex, need, geography	1.55	(1.24–1.95)	1.10	(0.91–1.32)	0.84	(0.69–1.02)	1.16	(1.01–1.34)	< 0.01
Private outpatient	SPR (95% CI)	Highest 2 quintiles	11.89	(10.94–12.91)	12.60	(11.88–13.35)	11.49	(10.86–12.14)	13.90	(13.33–14.49)	
		Lowest 2 quintiles	4.85	(4.31–5.42)	6.57	(6.07–7.10)	7.61	(7.13–8.11)	8.46	(8.03–8.91)	
		Rate difference	7.05		6.03		3.88		5.44		
		Rate ratio	2.45		1.92		1.51		1.64		
	RII (95% CI)	Adjusted to age, sex	7.04	(5.66–8.76)	3.41	(2.93–3.98)	1.99	(1.74–2.29)	2.61	(2.34–2.90)	< 0.01
		Adjusted to age, sex, need	7.29	(5.85–9.09)	3.56	(3.05–4.15)	2.06	(1.79–2.36)	2.74	(2.45–3.05)	< 0.01
		Adjusted to age, sex, need, geography	6.18	(4.88–7.82)	3.44	(2.94–4.03)	2.15	(1.87–2.48)	2.84	(2.54–3.17)	< 0.01
Public inpatient	SPR (95% CI)	Highest 2 quintiles	1.75	(1.40–2.17)	1.43	(1.20–1.69)	1.88	(1.63–2.15)	2.71	(2.46–2.97)	
		Lowest 2 quintiles	0.91	(0.68–1.18)	0.44	(0.33–0.59)	0.90	(0.74–1.08)	1.63	(1.44–1.83)	
		Rate difference	0.84		0.99		0.98		1.08		
		Rate ratio	1.93		3.22		2.09		1.66		
	RII (95% CI)	Adjusted to age, sex	4.02	(2.38–6.80)	4.93	(3.08–7.87)	2.74	(1.95–3.84)	2.38	(1.88–3.00)	< 0.01
		Adjusted to age, sex, need	4.04	(2.39–6.82)	5.01	(3.13–8.02)	2.81	(2.00–3.96)	2.48	(1.96–3.15)	< 0.01
		Adjusted to age, sex, need, geography	3.61	(2.05–6.34)	4.87	(3.02–7.88)	2.64	(1.86–3.75)	2.39	(1.88–3.03)	< 0.01
Private inpatient	SPR (95% CI)	Highest 2 quintiles	1.07	(0.80–1.40)	1.36	(1.13–1.62)	1.84	(1.59–2.11)	2.57	(2.33–2.84)	
		Lowest 2 quintiles	0.07	(0.02–0.16)	0.23	(0.15–0.34)	0.47	(0.36–0.60)	0.96	(0.82–1.12)	
		Rate difference	1.00		1.13		1.37		1.61		
		Rate ratio	15.33		5.87		3.94		2.68		
	RII (95% CI)	Adjusted to age, sex	80.44	(26.58–243.45)	17.92	(10.04–31.99)	9.24	(6.03–14.15)	6.98	(5.26–9.27)	< 0.01
		Adjusted to age, sex, need	78.99	(26.15–238.56)	18.21	(10.19–32.54)	9.57	(6.24–14.69)	7.23	(5.44–9.61)	< 0.01
		Adjusted to age, sex, need, geography	37.26	(11.81–117.60)	13.73	(7.66–24.62)	7.68	(4.97–11.88)	6.68	(5.02–8.90)	< 0.01

In the period 1993–2007, we found a continuing decrease in income-related inequalities in all types of healthcare, except public inpatient care in 2000. There was a larger decrease in the utilisation of public outpatient care by higher income groups compared with lower income groups. At the same time, utilisation of private outpatient care by lower income groups continued to increase, while utilisation by higher income groups remained stable. This led to the lower income-related inequalities in both public and private outpatient care utilisation. For public outpatient care utilisation, the fully adjusted RII decreased from RII 1.55 (95% CI: 1.24–1.95) in 1993 to RII 1.10 (95% CI: 0.91–1.02) in 2000, to RII 0.84 (95% CI: 0.69–1.02) in 2007 (Table 3). Private outpatient care utilisation showed a much sharper drop in income-related inequalities, with RII 6.18 (95% CI: 4.88–7.82) in 1993, to RII 3.44 (95% CI: 2.99–4.03) in 2000, to RII 2.15 (95% CI: 1.87–2.48) in 2007 (Table 3).

We observed increases in inpatient care utilisation for both higher and lower income groups in all types of healthcare. The increases were larger for lower income groups, which resulted in a sharp drop in inequalities, particularly in private inpatient care. Inequalities in private inpatient care utilisation decreased from RII 37.26 (95% CI: 1.81–117.60) in 1993, to RII 13.73 (95% CI: 7.66–24.62) in 2000, to RII 7.68 (95% CI: 4.97–11.88) in 2008 (Table 3).

In the period 2007–2014, we observed an increase in income-related inequalities in outpatient care utilisation and a continuing decrease in inequalities for inpatient care utilisation. The prevalence rates for public and private outpatient care utilisation increased for both higher and lower income groups. However, the increases were larger for higher income groups, which resulted in larger inequalities in public and private outpatient care utilisation in 2014 compared with 2007. For example, income-related inequalities in private outpatient care utilisation increased from RII 2.15 (95% CI: 1.87–2.48) in 2007 to RII 2.84 (95% CI: 2.54–3.17) in 2014 (Table 3).

We observed a continuing decrease in inequalities in public and private inpatient care utilisation in 2014, although the decreases were much smaller compared with the period 1993–2007. For example, income-related inequalities in private inpatient care utilisation decreased from RII 7.68 (95% CI: 4.97–11.88) in 2007 to RII 6.68 (95% CI: 5.02–8.90) in 2014 (Table 3) compared with the period 2000–2007, when this decreased from RII 13.73 (95% CI: 7.66–24.62) to RII 7.68 (95% CI: 4.97–11.88).

Estimates of income-related inequalities in overall utilisation are displayed in Table 4. For overall outpatient care utilisation, income-related inequalities decreased from 1993 (RII 3.31; 95% CI: 2.78–3.94) to 2000 (RII 2.33; 95% CI: 2.05–2.64) and even further until 2007, (RII 1.58; 95% CI: 1.40–1.79). A subsequent increase was observed for 2014 (RII 2.16; 95% CI: 1.97–2.38). A similar trend is observed in income-related inequalities in both public and private outpatient care. For overall inpatient care utilisation, there was an increase in income-related inequalities from 1993 (RII 6.26; 95% CI: 3.80–10.31) to 2000 (RII 7.63; 95% CI: 5.25–11.08), but a subsequent decrease in 2007 (RII 4.02; 95% CI: 3.06–5.29) and 2014 (RII 3.68; 95% CI: 3.05–4.43). The pattern of income-related inequalities in overall utilisation of inpatient care is similar to the pattern of inequalities in the utilisation of public inpatient care, but different with the pattern of inequalities in the utilisation of private inpatient care which showed a continuing decrease from 1993 to 2014.

**Table 4:** Trends in absolute and relative income-related inequalities in overall utilisation, 1993–2014.

			1993		2000		2007		2014		p-value
Overall outpatient	SPR (95%CI)	Highest 2 quintiles	21.10	(19.82–22.44)	18.11	(17.24–19.01)	15.72	(14.98–16.48)	19.20	(18.53–19.89)	
		Lowest 2 quintiles	11.54	(10.73–12.41)	11.83	(11.15–12.53)	12.09	(11.48–12.72)	13.57	(13.02–14.13)	
		Rate difference	9.55		6.28		3.63		5.63		
		Rate ratio	1.83		1.53		1.30		1.42		
	RII (95%CI)	Adjusted to age, sex	3.36	(2.86–3.94)	2.32	(2.05–2.63)	1.56	(1.39–1.75)	2.04	(1.87–2.24)	0.32
		Adjusted to age, sex, need	3.54	(3.01–4.17)	2.42	(2.13–2.75)	1.61	(1.43–1.81)	2.16	(1.97–2.37)	<0.01
		Adjusted to age, sex, need, geography	3.31	(2.78–3.94)	2.33	(2.05–2.65)	1.58	(1.40–1.79)	2.16	(1.97–2.38)	< 0.01
Overall inpatient	SPR (95%CI)	Highest 2 quintiles	2.83	(2.38–3.34)	2.72	(2.39–3.07)	3.62	(3.27–3.99)	5.08	(4.74–5.45)	
		Lowest 2 quintiles	0.99	(0.76–1.28)	0.66	(0.51–0.83)	1.36	(1.16–1.58)	2.56	(2.32–2.81)	
		Rate difference	1.84		2.06		2.26		2.53		
		Rate ratio	2.86		4.13		2.67		1.99		
	RII (95%CI)	Adjusted to age, sex	8.20	(5.14–13.09)	8.61	(5.97–12.42)	4.45	(3.41–5.81)	3.73	(3.10–4.48)	<0.01
		Adjusted to age, sex, need	8.22	(5.15–13.11)	8.77	(6.07–12.67)	4.61	(3.53–6.04)	3.90	(3.24–4.69)	<0.01
		Adjusted to age, sex, need, geography	6.26	(3.80–10.31)	7.63	(5.25–11.08)	4.02	(3.06–5.29)	3.68	(3.05–4.43)	<0.01

**Fig 3:**
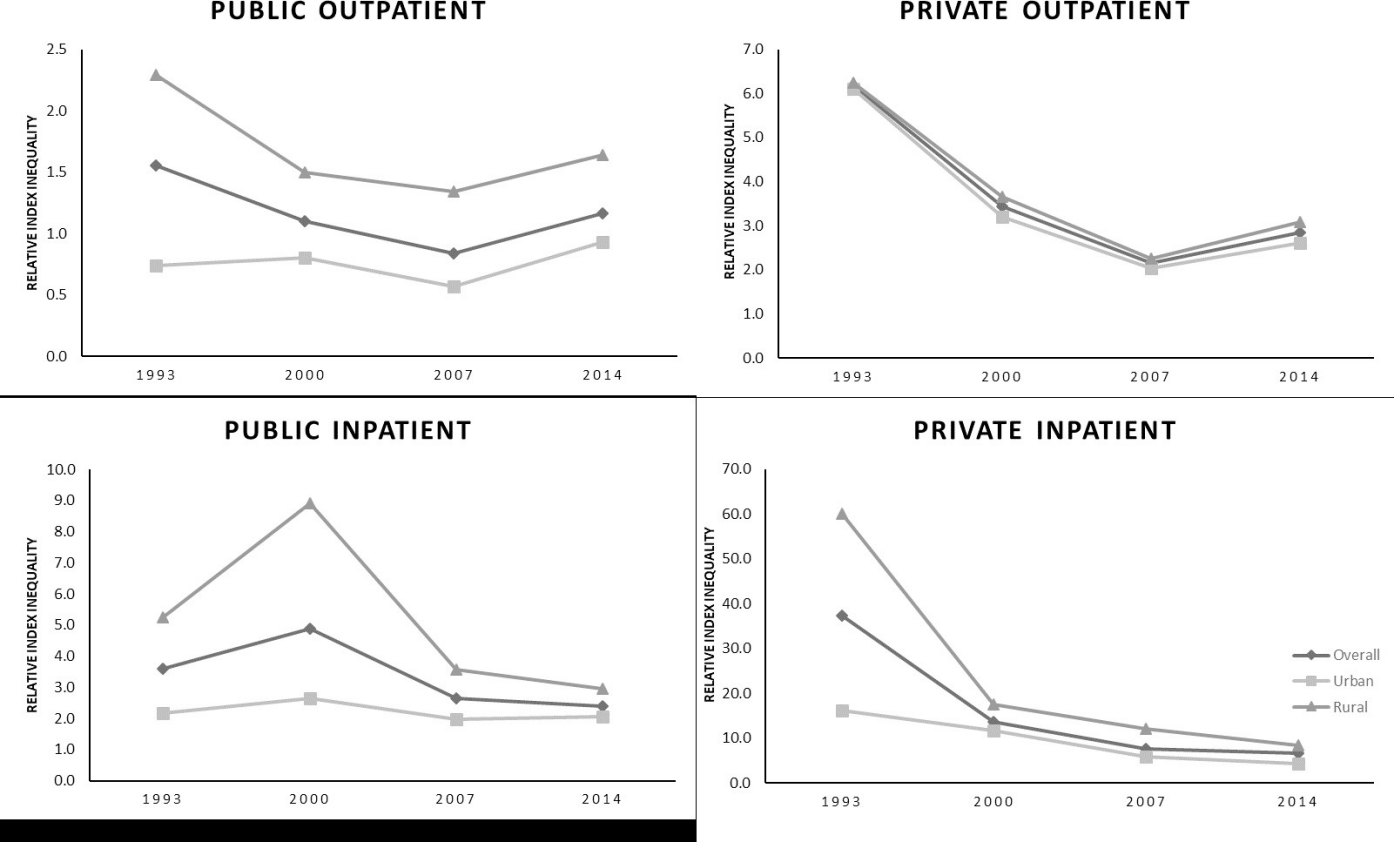
Trends of income-related inequalities in various type of healthcare utilisation by urban-rural area, 1993–2014.

Trends of income-related inequalities in healthcare utilisation according to urban versus rural area of residence are given in Fig 3. The pattern of income-related inequalities in different types of healthcare utilisation was similar between urban and rural areas. However, inequalities were generally larger in rural areas than urban areas, for most type of healthcare utilisations, except for private outpatient care utilisation.

## Discussion

We aimed to describe the trends in income-related inequalities in the utilisation of public outpatient care, private outpatient care, public inpatient care, and private inpatient care as well as the overall utilisation in Indonesia during the period 1993–2014. We found relatively large inequalities in the utilisation of private outpatient care, public inpatient care, and private inpatient care in the early 1990s. However, the pattern of inequalities over the last two decades varies between different type of healthcare. Although inequalities continued to decrease during the period 1993–2007, inequalities in public and private outpatient care increased in 2014. Inequalities in public and private inpatient care continued to decrease during the period 1993–2014, with a particularly large decrease observed for private inpatient care.

To the best of our knowledge, ours is the first study to use a historical perspective with a relatively long observation period to better understand the changes of income-related inequalities in healthcare in Indonesia over time. Our study used the same dataset during different periods, which minimised the problem of measurement and data comparability. The dataset used in our study was based on a nationally representative survey with a high response rate (above 90% in four periods of the survey) [18].

A possible limitation of our study is the measurement of healthcare need, which was restricted to SAH. The prevalence of diseases and their pattern among different population groups changed over the last two decades, and such changes likely contributed to the changes in the healthcare utilisation. Ideally, we would have included disease-based need indicators to our analysis and/or provided inequality estimation for disease-specific healthcare utilisation. However, in our dataset, most of the individuals’ disease status was only measured using self-reported measurement which is often problematic for use in the inequality measurement in the LMICs’ setting such as Indonesia [22]. Moreover, standardised measures of diseases were available in our dataset only in the last two survey years (2007 and 2014) which made it incomparable to include into the analysis for the entire study period.

Another limitation is related to the use of self-reported healthcare utilisation, as such healthcare utilisation measures may be subject to recall bias. However, this was our only option, because no national registry-based healthcare utilisation data were available in Indonesia. Previous studies in Indonesia also used this self-reported data from the Indonesian Ministry of Health and Central Bureau of Statistics [23, 24].

The RII captures individual differences in the socioeconomic position [21], and in our case, a large RII may be the result of larger differences in the income between individuals as well as larger differences in the utilisation of the healthcare between different individuals in various income levels. Our findings showed that the differences in income levels between income quintiles decreased over time, which implied that this component could not explain the increase of income-related differences in health care utilisation.

We discuss our findings within the context of changes to the Indonesian healthcare system during the period 1993–2014, which we have summarised in Table 1. A summary of trends in demand and supply side factors in the period of 1993 to 2014 is given in Table 5. A combination of several demand-supply factors can explain the relatively large observed levels of inequalities in the utilisation of private outpatient care, public inpatient care, and private inpatient care in 1993. First, in the early 1990s, the main focus of government was public health intervention particularly for communicable diseases [15]. Access to personal healthcare become an individual responsibility and was regulated by the market mechanism. Only higher income groups with sufficient financial resources could generally afford healthcare [3, 16].

**Table 5:** Trends of demand and supply-side factors in healthcare utilisation in Indonesia, 1993–2014.

	1993	2000	2007	2014
Health insurance coverage for total population (%)	13.8	21.3	50.1	52.5
Physician population ratio (per 100,000)	9.4	11.0	19.9	16.2
Public primary care facilities population ratio (per 100,000)	3.3	3.6	3.7	3.9
Hospital beds population ratio (per 100,000)	60.8	61.0	63.3	107.0
Median costs of healthcare^1^				
Public outpatient (per visit)	1.1	4.0	8.0	20.0
Private outpatient (per visit)	11.0	25.0	40.0	60.0
Public inpatient (per admission)	148.5	600.0	1,200.0	2,300.0
Private inpatient (per admission)	568.7	1,170.0	2,500.0	3,500.0

Source: Indonesia’s health profile (various years), IFLS data; ^1^In thousand Indonesian Rupiah (unadjusted).

Second, the majority of the population has no insurance coverage, and coverage of government health insurance was limited to civil servants [15], which is reflected in a low coverage of health insurance in the total population (see Table 5). A lack of health insurance coverage was a significant financial barrier to healthcare for lower income groups [25].

Third, the cost of private outpatient care and public and private inpatient care was relatively high (Table 5). For example, median costs of public inpatient care per admission were larger than the median monthly income for the richest income quintile. For inpatient care, although public hospitals were the primary providers of healthcare, the government provided only limited subsidies, which made this healthcare relatively unaffordable for lower income groups. Fourth, the supply of private inpatient care was very limited because capacity was very low and private hospitals were located in big cities. The cost of private inpatient care was even higher than public inpatient care [26]. Consequently, lower income groups had minimal access to private inpatient care.

Our findings show that inequalities generally decreased during the period 1993–2007. More specifically, utilisation continually increased, particularly for private outpatient care and private inpatient care among lower income groups. The expansion of government health insurance programmes for the poor that started with JPS-BK (1997), ASKESKIN (1999), and JAMKESMAS (2004) has led to a sharp increase in the health insurance coverage between 2000 and 2007 (Table 5). This may have increased healthcare utilisation among lower income groups and contributed to the reduction of inequalities. A panel study using data from national economic surveys in 2005 and 2006 showed that the ASKESKIN and JAMKESMAS programmes were effective, and increased healthcare utilisation for the poor, particularly for outpatient care [17]. A report from the World Bank based on data from the 2010 national economic survey showed that JAMKESMAS beneficiaries utilised outpatient and inpatient care more than beneficiaries of government health insurance for civil servants and private health insurance [16].

During the same period, we observed increased utilisation of private healthcare among lower income groups. This finding may be related to health sector decentralisation, which started in 2001. Decentralisation provided district governments with the full authority to restructure the provision of healthcare in their districts. One of the most prominent features of this restructuring process is the greater involvement of private healthcare facilities (privatisation). The number of private healthcare providers increased considerably after decentralisation. Because the JAMKESMAS programme contracted them, these private providers played a greater role in providing healthcare for lower income groups [15, 16, 27].

We found an increase in income-related inequalities in public and private outpatient care utilisation during the period 2007–2014. This finding was unexpected, considering previous trends of decreasing inequality, and the increase of health insurance coverage during this period. However, a recent study had shown that income-related inequalities in public and private outpatient care increased after the introduction of the NHI programme when utilisation was adjusted for healthcare needs [14]. There are several plausible explanations for this phenomenon. First, a 2010 report using national data showed there had been problems with the identification and enrolment of JAMKESMAS beneficiaries, which meant that about 20% of the beneficiaries belonged to higher income groups [16]. These ‘pseudo’ JAMKESMAS beneficiaries have better resources (e.g. financial ability and health knowledge), which enable them to use more healthcare compared with the ‘real’ beneficiaries from lower income groups.

Second, the undersupply and unequal distribution of the healthcare workforce may explain the increase in inequalities. The growing undersupply is indicated by the decrease in the physician population ratio and an only modest increase in public primary care availability (Table 5). This healthcare shortage resulted in other access problems (such as longer waiting times or waiting lists for utilising healthcare), which increased other forms of financial barriers such as opportunity costs. Loss of productivity due to the time needed to utilise healthcare affected lower income groups more than higher income groups.

From the perspective of healthcare providers, the JAMKESMAS programme had a low-profit-margin, which provided little incentive to increase the healthcare supply. Many healthcare providers offered more exclusive healthcare options (e.g. bypassing waiting lists) at a higher price to generate more revenue and compensate for the JAMKESMAS programme’s low reimbursement fees [15]. These kinds of services can only be accessed by people in higher income groups with sufficient financial resources.

Third, our findings showed that the RII for public outpatient care decreased after adjusting for geographical factors. We argue that the unequal distribution of healthcare services may explain the widening inequalities in 2014, particularly in public outpatient care. Most JAMKESMAS beneficiaries use public healthcare facilities to access healthcare, and the data showed disparities between rural and urban areas in the availability of public healthcare facilities [14]. However, we cannot apply this argument to explain widening inequalities in private outpatient care utilisation, because our findings showed that RII did not decrease, and even increased, after adjusting for geographical factors. Considering the nature of private healthcare–which is more expensive compared with public healthcare, and is still dominated by out-of-pocket payments–we argue that financial ability may have played a bigger role.

Findings from our study also show that although inequalities in private inpatient care continue to decrease over time in relative terms, absolute inequalities are increasing. This reduction in relative inequalities might be attributable to the growing role of private healthcare facilities. The number of private hospitals was substantially increased in the last few years as showed by data from the Ministry of Health in 2014 that 64.4% of the hospitals in Indonesia are private hospital [15]. The government contracts many of these private hospitals to provide healthcare for ASKESKIN and JAMKESMAS beneficiaries, which makes private inpatient care more accessible to lower income groups. However, even though private inpatient care is becoming more accessible to lower income groups and increasing their utilisation, utilisation of private inpatient care is increasing to an even greater extent among higher income groups, which is likely due to their financial advantage. This provides a probable explanation for the larger absolute inequality in private inpatient care.

## Conclusions

Our study demonstrates trends in income-related inequalities in healthcare utilisation in Indonesia over the last two decades. Our findings show that in the later period, income-related inequalities in healthcare utilisation may have increased, although health insurance coverage continues to increase. Several changes to the healthcare system (such as the adequacy and distribution of healthcare, and health sector decentralisation) provide additional explanations for how income-related inequalities in Indonesia have changed over time.

Our study underlines that the current health policy to expand the NHI programme can be improved by better targeting health insurance coverage to reduce income-related inequalities in healthcare. However, it is also important to balance efforts to expand health insurance coverage with more investments to improve the healthcare supply and distribution. This could include greater public investment in healthcare facilities, better regulation of the distribution of healthcare personnel, and maximising the role of private providers to address healthcare demands. A systematic monitoring and evaluation of those policies is critical to prevent the further increase of income-related inequalities in healthcare utilisation in Indonesia.

## Supporting information

S1 Table Association between food consumption and other consumption in IFLS 5 (2014). (PDF)

S2 Table Prevalence rate of healthcare utilisation for all type of healthcare, for total population and by income quintiles, 1993–2014. (PDF)
