# Supplementary material for: The evolution of income-related inequalities in healthcare utilisation in Indonesia, 1993–2014
Source: PLoS One. 2019 Jun 25;14(6):e0218519. doi: 10.1371/journal.pone.0218519 (PMC6592526; doi:10.1371/journal.pone.0218519)
Supplement: S1 Table — (PDF) [file pone.0218519.s001.pdf]

S1 Table. Association between food consumption and other consumption in IFLS 5 (2014).

		p-value
**Correlation (r)**		
Food consumption to non-food consumption	0.553	0.00
Food consumption to total consumption	0.708	0.00
**Proportion of food consumption to total consumption (%)**		
Quintile 1 (poorest)	23.4	
Quintile 2	22.8	
Quintile 3	21.9	0.00
Quintile 4	20.1	
Quintile 5 (richest)	14.2	
