# Supplementary material for: The evolution of income-related inequalities in healthcare utilisation in Indonesia, 1993–2014
Source: PLoS One. 2019 Jun 25;14(6):e0218519. doi: 10.1371/journal.pone.0218519 (PMC6592526; doi:10.1371/journal.pone.0218519)
Supplement: S2 Table — (PDF) [file pone.0218519.s002.pdf]

S2 Table. Prevalance rate of healthcare utilisation for all type healthcare, for total population and by income quintiles, 1993-2014

	1993		2000		2007		2014	
	UPR[a]	SPR[b]	UPR[a]	SPR[b]	UPR[a]	SPR[b]	UPR[a]	SPR[b]
**Overall outpatient**								
Total	15.9	-	14.4	-	13.6	-	16.3	-
Quintile 1	10.8	10.8	10.2	10.2	11.9	11.8	12.8	12.5
Quintile 2	12.8	12.9	12.6	12.7	12.0	12.1	14.9	14.7
Quintile 3	15.9	16.0	14.7	14.6	14.0	14.0	16.3	16.4
Quintile 4	19.8	19.9	16.1	16.0	14.7	14.7	17.7	15.9
Quintile 5	22.9	23.2	18.8	18.9	15.7	15.9	19.7	20.1
**Public outpatient**								
Total	8.3	-	5.8	-	4.7	-	6.2	-
Quintile 1	7.5	7.5	5.2	5.2	4.7	4.6	6.2	6.1
Quintile 2	7.2	7.2	5.7	5.8	4.6	4.7	6.1	6.0
Quintile 3	9.0	9.0	6.1	6.1	5.0	5.0	5.7	5.8
Quintile 4	9.2	9.3	6.3	6.3	4.9	4.9	6.7	7.0
Quintile 5	8.7	8.9	5.7	5.8	4.0	4.1	6.2	6.4
**Private outpatient**								
Total	8.4	-	9.2	-	9.3	-	11.2	-
Quintile 1	3.6	3.6	5.6	5.6	7.4	7.4	7.5	7.2
Quintile 2	6.2	6.2	7.2	7.2	7.7	7.7	9.8	9.7
Quintile 3	7.7	7.8	9.2	9.1	9.3	9.3	11.6	11.7
Quintile 4	11.7	11.4	10.4	10.4	10.2	10.2	12.3	12.6
Quintile 5	12.4	12.4	14.1	14.2	12.2	12.3	15.0	15.1
**Overall inpatient**								
Total	1.8	-	1.7	-	2.4	-	2.1	-
Quintile 1	0.6	0.7	0.7	0.7	1.6	1.6	1.6	2.2
Quintile 2	1.3	1.3	0.7	0.7	1.2	1.2	1.8	2.8
Quintile 3	1.7	1.7	1.6	1.6	2.3	2.3	1.8	3.1
Quintile 4	1.9	2.0	2.0	2.0	3.0	3.0	2.3	4.0
Quintile 5	3.9	3.9	3.5	3.5	4.2	4.2	2.9	6.1
**Public inpatient**								
Total	1.3	-	0.9	-	1.4	-	2.1	-
Quintile 1	0.6	0.7	0.5	0.5	1.1	1.1	1.6	1.5
Quintile 2	1.1	1.1	0.4	0.4	0.8	0.7	1.8	1.8
Quintile 3	1.3	1.3	0.9	0.9	1.6	1.6	1.8	1.9
Quintile 4	1.3	1.3	1.3	1.3	1.6	1.6	2.3	2.4
Quintile 5	2.3	2.3	1.6	1.6	2.1	2.1	2.9	3.0
**Private inpatient**								
Total	0.5	-	0.8	-	1.0	-	1.6	-
Quintile 1	0.0	0.0	0.2	0.2	0.5	0.5	0.8	0.8
Quintile 2	0.2	0.2	0.3	0.3	0.5	0.5	1.1	1.1
Quintile 3	0.4	0.4	0.7	0.7	0.7	0.7	1.2	1.2
Quintile 4	0.6	0.7	0.8	0.8	1.5	1.5	1.7	1.8
Quintile 5	1.6	1.6	1.9	2.0	2.2	2.2	3.2	3.4

[a]Unstandardised prevalance rate per 100 person, [b]Age-sex standardised prevalence rate per 100 persons
